# BSA-Stabilized Mesoporous Organosilica Nanoparticles Reversed Chemotherapy Resistance of Anaplastic Thyroid Cancer by Increasing Drug Uptake and Reducing Cellular Efflux

**DOI:** 10.3389/fmolb.2020.610084

**Published:** 2020-12-03

**Authors:** Xiao Han, Xiaoquan Xu, Yuxia Tang, Feipeng Zhu, Ying Tian, Wei Liu, Doudou He, Guangming Lu, Yunfei Gu, Shouju Wang

**Affiliations:** ^1^The First School of Clinical Medicine, Nanjing University of Chinese Medicine, Nanjing, China; ^2^Department of Radiology, Jinling Hospital, Nanjing Medical University, Nanjing, China; ^3^Department of Radiology, The First Affiliated Hospital of Nanjing Medical University, Nanjing, China

**Keywords:** anaplastic thyroid cancer, chemotherapy resistance, bovine serum albumin, drug efflux, organosilica

## Abstract

Anaplastic thyroid cancer (ATC) is a highly aggressive and the most lethal type of thyroid cancer. The standard-of-care for unresectable ATC is radiotherapy and chemotherapy, usually based on doxorubicin (Dox). However, most patients develop resistance shortly after treatment. To overcome the drug resistance, we synthesized the mesoporous organosilica nanoparticles (MONPs) loaded with Dox and stabilized the nanocomposites by bovine serum albumin (BSA). The surface area and pore volume of MONPs were 612.653 m^2^/g and 0.589 cm^3^/g. The loading capacity of Dox-MONPs reached 47.02%. Compared to Dox-MONPs and free Dox, BSA-Dox-MONPs had more durable tumor-killing power on both drug-sensitive cell line HTh74 and drug-resistant cell line HTh74R. The cellular uptake of BSA-Dox-MONPs was 28.14 and 65.53% higher than that of Dox-MONP in HTh74 and HTh74R. Furthermore, the BSA coating decreased the efflux rate of nanocomposites in HTh74 (from 38.95 to 33.05%) and HTh74R (from 43.03 to 32.07%). In summary, BSA-Dox-MONPs reversed the chemotherapy resistance of ATC cells via increased drug uptake and inhibited drug efflux, offering a promising platform for the treatment of chemo-resistant ATC.

## Introduction

Anaplastic thyroid cancer (ATC) has lately received considerable attention for reduced median survival rate and high invasion. The median survival of patients is no longer than 5 months, and the two-year survival rate is less than 15% (Molinaro et al., 2017). Combination treatment, including extensive resection and adjuvant chemo-radiotherapy, is recommended (Haddad et al., 2018). Although doxorubicin (Dox) is the only chemo-drug suggested, the resistance to Dox is collective in ATC, leading to a worse prognosis (Haddad et al., 2018). A critical mechanism behind the Dox resistance is the excessive efflux of chemotherapy drugs (Zinzi et al., 2014; Davis et al., 2015). Thus, developing new strategies to overcome chemoresistance is crucial to improve patients’ outcomes with ATC.

Nowadays, mesoporous nanoparticles have attracted extensive interest because their high loading capacity is suitable for drug loading and delivery (Yao et al., 2017; Teng et al., 2018; Chen et al., 2019, 2020). Among these mesoporous materials, considerable attention has been paid to mesoporous organosilica nanocapsules (MONPs) since their excellent biocompatibility, large surface area, adjustable pore volume, and easily modified surface (Tao et al., 2018; Teng et al., 2018; Yao et al., 2020). MONPs are synthesized using organic group-bridged siloxanes as silicon sources, which is different from pure Si-O-Si groups of inorganic mesoporous silica nanoparticles (MSNs) (Chen et al., 2013; Teng et al., 2014b; Croissant et al., 2015). These doped organic groups give MONPs more advantages, including improved hydrothermal stability and dispersibility (Chen et al., 2014). Furthermore, MONPs can be modified more easily than MSNs (Yu et al., 2011; Burglova et al., 2014). By selectively introducing organic groups, the hydrophilicity of the pores can be adjusted for controllable drug loading and release (Chen et al., 2014; Croissant et al., 2014; Wu et al., 2016). Moreover, MONPs have higher blood compatibility due to attenuated interaction between Si-OH and red blood cells. The previous studies have shown that MONPs exhibit significantly lower hemolysis than MSNs (Yu et al., 2011).

Recently, bovine serum albumin (BSA) has been widely applied in the drug delivery system through coupling interaction on account of its low cytotoxicity, minor immunogenicity, and excellent biocompatibility (Liu et al., 2016; Fang et al., 2019; Pan et al., 2020; Wu et al., 2020). Meanwhile, BSA can improve the stealthiness of the nanoparticles in the blood circulation, thereby enhancing tumor-specific accumulation (Fang et al., 2019). Zhang’s research showed that drug-loaded MSNs are more stable and can maintain excellent dispersibility in serum after coated with BSA, leading to accumulation in tumor sites by enhanced permeation and retention (EPR) effect (Zhang et al., 2019). Because of the high metabolism rate, cancer cells need more nutrients and internalized a greater amount of proteins (such as BSA) and amino acids than normal cells. Therefore the BSA coating results in increased cancer cell internalization (Deberardinis and Chandel, 2016). Several studies have shown that BSA modified Au-NPs and galactosylated nanoparticles have greater tumor uptake; however, the effect of BSA coating on MONPs has been scarcely reported (Huang et al., 2018; Unnikrishnan et al., 2020). In our research, BSA-stabilized MONPs were studied to be quantified not only for cellular uptake but also for cellular efflux in ATC.

Currently, there are few nanoplatforms developed for drug-resistant ATC (Marano et al., 2016, 2017; Wang et al., 2018). In our previous research, we constructed Dox-loaded melanin nanoparticles to enhance the efficacy of chemotherapy to drug-resistant ATC (Wang et al., 2018). However, the Dox-loading capacity of melanin nanoparticles was less than 20%, which may be related to their limited surface-area-to-volume ratio (Wang et al., 2018). To increase the loading capacity, herein, we synthesized MONPs (Teng et al., 2014a) to replace melanin nanoparticles for Dox loading. The mesoporous structure and Dox-loading capacity of MONPs were characterized. In addition, BSA coating was used to stabilize Dox-MONPs in chemotherapy-resistant ATC research. The therapeutic efficacy, as well as cellular uptake and efflux of BSA-Dox-MONPs, was compared with Dox-MONPs in drug-sensitive and drug-resistant ATC.

## Materials and Methods

### Chemicals and Materials

Cetyltrimethylammonium bromide (CTAB), concentrated aqueous ammonia solution (25–28 wt%), anhydrous ethanol, Dox, and dimethyl sulfoxide (DMSO) were obtained from Aladdin Reagent. Tetraethoxysilane (TEOS), 1,4-Bis(triethoxysilyl)propane tetrasulfide (TESPTS), and 3-(4,5-Dimethylthiazol-2-yl)-2,5-diphenyltetrazolium bromide (MTT) were purchased from Sigma-Aldrich (St. Louis, MO, United States). BSA was supplied by the Biosharp company. Deionized water (Millipore) with a resistivity of 18.2 MΩ⋅cm was used in all experiments. 4,6-diamino-2-phenyl indole (DAPI) was provided by Nanjing Keygen Biotech. Co., Ltd. (Nanjing, China). Cancer cells were cultured in F-12 medium with 1% amphotericin, 1% 500x penicillin and streptomycin, and 10% fetal bovine serum (FBS), which were offered by Gibco company. Gibco company offered DMEM medium used to culture HaCaT cells and phosphate buffer saline (PBS) for cell experiments. YF488-AnnexinV was purchased from United States Everbright^®^Inc.

### Synthesis of Mesoporous Organosilica Nanoparticles

Mesoporous organosilica nanoparticles (MONPs) were synthesized according to previously reported protocols (Teng et al., 2014a). Briefly, 0.16 g CTAB was dissolved in a mixture of 75 mL deionized water and 30 mL ethanol containing 1 mL concentrated aqueous ammonia solution (25 wt%). After being vortexed vigorously at 35°C for one-hour, a mixture of TEOS (0.1 mL) and TESPTS (0.25 mL) was then quickly added into the solution, and the reaction was allowed to proceed for 24 h. The product was precipitated by centrifugation and washed thrice with ethanol. Then, the product was resuspended in 37% hydrochloric acid solution in ethanol at a ratio of 1:500 (v/v), gently vortexed at 60°C for 3 h, and collected by centrifugation for three times. Finally, the product was rewashed in pure ethanol thrice and dried in a high vacuum.

### Loading Dox to MONPs

Mesoporous Organosilica Nanoparticles stock suspension at a final concentration of 1 mg/mL was prepared by adding MONPs powder to deionized water. To load MONPs with Dox, the MONPs stock suspension was mixed with various concentrations of Dox aqueous solution and vortexed vigorously in the dark for 24 h at room temperature. To find the optimal MONPs: Dox ratio that maximizes the loading efficiency and loading capacity, a series of Dox: MONPs suspension with mass ratio from 0.125: 1 to 8: 1 was set. The Dox concentration in the supernatant was determined by measuring the UV absorbance and comparing it to the standard curve. The loading efficiency and loading capacity were calculated based on the free Dox concentration.

### Coating BSA on Dox-MONPs

Dox-MONPs aqueous solution with a concentration of 200 μg/mL was added dropwise into the BSA aqueous solution with a concentration of 1 mg/mL, which was gently vortexed at physiological temperature 37°C. After the addition, the mixture was gently vortexed at 37°C for 3 h, followed by centrifugation and redispersion.

### Characterization of Nanoparticles

The UV-Vis spectra of MONPs and its modified products were obtained using PerkinElmer Lambda 35 UV-Vis spectrophotometer. Its hydrodynamic size and Zeta potentials were obtained from a Brookhaven Zeta PALS machine. The transmission electron microscope (TEM) photograph was taken by an FEI TECNAI F20s TWIN microscope. A high angle annular dark-field (HAADF) scanning TEM and energy dispersive X-ray (EDX) analyses were performed (FEI TECNAI F20s TWIN) to characterize the morphologies of nanomaterials and the distribution of organic elements. The samples were suspended in ethanol ultrasonically and supported onto an ultrathin carbon-coated tinned grid for TEM and EDX measurements. Nitrogen adsorption and desorption experiments were used to measure the pore size and uniformity of MONPs.

### MTT Assay

HTh74 and HTh74R cells were routinely cultivated in 25 cm^2^ culture flasks at 37°C, 5% CO_2_, and 95% humidity, using McCoy F12 medium supplemented with 10% FBS, 1% penicillin and streptomycin. For MTT assay, HTh74 and HTh74R cells at the logarithmic growth phase were seeded in 96-well plates and cultured for 24 h. Different concentrations of free Dox, Dox-MONPs, and BSA-Dox-MONPs were added to 96-well plates and incubated with HTh74 and HTh74R cells for another 24 h. Afterward, the drug-containing culture medium was aspirated and replaced with a complete culture solution containing 0.5% MTT. After 4 h, the formazan produced was dispersed in DMSO and shaken for 10 min to promote sufficient dissolution. Cell viability was quantified by the absorbance at 570 nm with the absorbance at 630 nm as reference. Human Keratinocyte Cells (HaCaT) were cultured in DMEM medium supplemented with 10% FBS, 1% penicillin and streptomycin, at 37°C, 5% CO_2_, and 95% humidity. After incubating HaCat with various concentrations of MONPs for 24 h, MTT assays were performed as described above.

### Confocal and Fluorescence Microscope Image Analysis

For fluorescence morphology, HTh74 and HTh74R cells were co-incubated with Dox-MONPs and BSA-Dox-MONPs with an equivalent Dox concentration of 25 μg/mL for 6 h and washed gently in cold PBS thrice. Cells obtained were stained by DAPI dye following the instructions and washed twice with PBS. Afterward, the samples were imaged on a confocal laser scanning microscope.

In another experiment, HTh74 and HTh74R cells were first incubated in 12-well plates for 24 h and further incubated with Dox-MONPs and BSA-Dox-MONPs at an equivalent Dox concentration of 25 μg/mL for another 6 h. After incubation, the treated cells were gently washed three times with PBS and further stained with YF488-AnnexinV in the dark according to the instructions. Then the cells obtained were visualized and photographed by fluorescence microscopy.

### Flow Cytometry Test

To compare the uptake of Dox-MONPs and BSA-Dox-MONPs, the HTh74 and HTh74R were incubated with Dox-MONPs and BSA-Dox-MONPs containing 25 μg/mL of equivalent effective Dox for 6 h, following with PBS rinses thrice. Then the cells were collected and digested into single cells, subjected to flow cytometry, and the Dox fluorescence signals were measured by Cytoflow cytometer.

In another experiment, HTh74 and HTh74R cells were first cultured in media containing BSA-Dox-MONPs or Dox-MONPs with an equivalent Dox concentration of 25 μg/mL. After 6 h, the nanoparticle-containing media were removed, and the cells were cultured in fresh media for an additional 18 h to allow the efflux of nanoparticles. The cell fluorescence signals before and after culture in fresh media were recorded to evaluate the efflux of nanomaterials. The formula for the efflux ratio of drug-loaded nanoparticles is (F_1_-F_2_)/F_1_ × 100%, where F_1_ represents the average intracellular fluorescence intensity before culture in fresh media, and F_2_ indicates the average intracellular fluorescence intensity after culture in fresh media.

### Statistical Analysis

All data were analyzed using Graphpad Prism 7.0 software. All quantitative data are expressed as mean ± SD and analyzed using the one-way ANOVA or *t*-test. The value of 0.05 was defined as a statistically significant threshold.

## Results and Discussion

**FIGURE 1:**
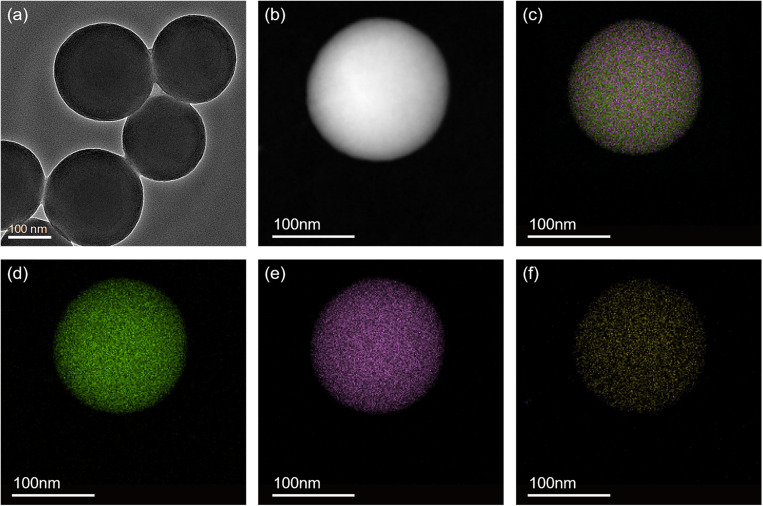
TEM image of **(A)** MONPs and HADDF image of **(B)** MONPs. EDX elemental mapping images of the corresponding MONPs: **(C)** merge of O, Si and S elements, **(D)** oxygen, **(E)** silicon and **(F)** sulfur.

Mesoporous Organosilica Nanoparticles were successfully synthesized and characterized. TEM image results show that the MONPs are spherical with an average diameter of 224.3 ± 21.2 nm. Notably, the EDX elemental mapping results show that the Si, O, and S elements are uniformly distributed, demonstrating the S elements were successfully doped in the organosilica frameworks (Figure 1).

**FIGURE 2:**
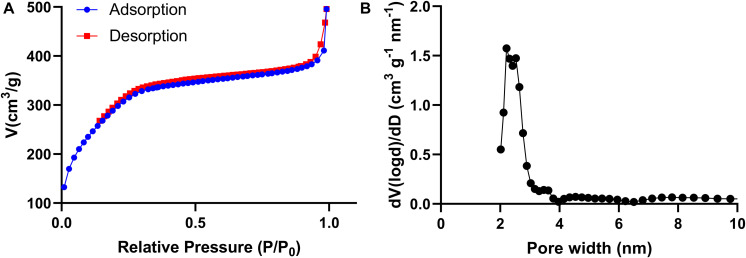
**(A)** Nitrogen adsorption-desorption isotherms and **(B)** pore-size distribution curves of the MONPs.

The nitrogen adsorption-desorption isothermal curve conforms to the type IV curve, revealing the typical characteristics of MONPs with uniform mesopore size distribution. The surface area and pore volume of MONPs are 612.653 m^2^/g and 0.589 cm^3^/g, respectively. The pore size distribution was calculated to be peaked at 2.2 nm according to the NLDFT method (Figure 2).

**FIGURE 3:**
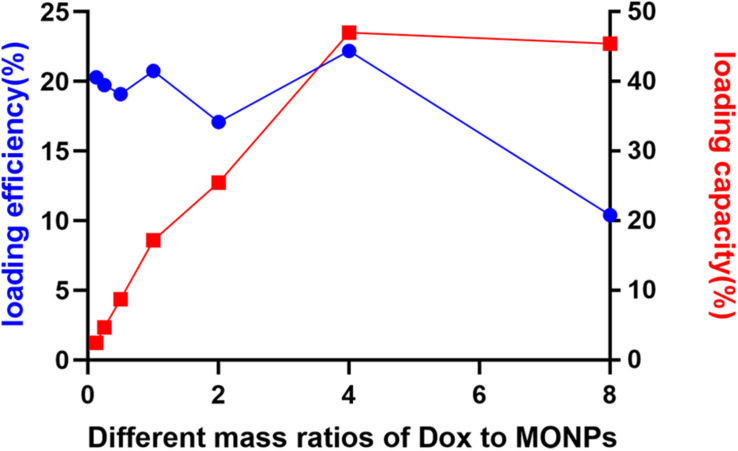
Loading efficiency and loading capacity of Dox-MONPs at different mass ratios of Dox to MONPs.

In order to quantitatively evaluate the Dox loading efficiency and capacity of MONPs, a constant amount of MONPs was incubated with various concentrations of Dox. As the Dox-to-MONPs ratio increases, the loading capacity increases and plateaus to about 45% when the mass ratio is equal or greater than four (Figure 3 and Supplementary Figures 1, 2). However, the Dox loading efficiency fluctuates around 20% and drops when the Dox concentration is high. Taking account of both loading efficiency and capacity, the mass ratio of Dox-to-MONPs was set at 4:1 for the following experiments.

**FIGURE 4:**
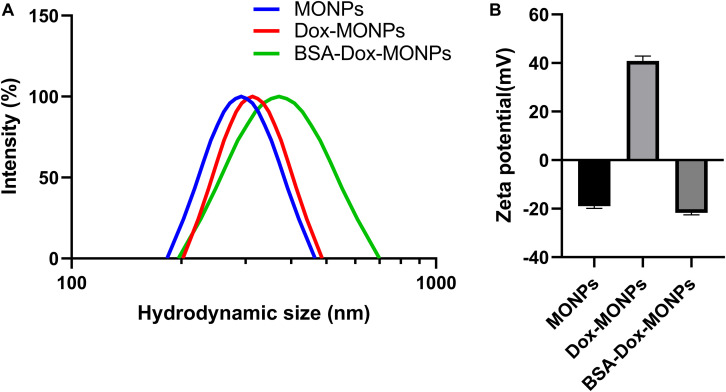
**(A)** The hydrodynamic size and **(B)** Zeta potential of MONPs, Dox-MONPs and BSA-Dox-MONPs.

To stabilize MONPs for biomedical applications, the obtained Dox-MONPs were vortexed with BSA solutions. The hydration diameter of MONPs was 291.92 ± 11.85 nm, which was slightly larger than the diameter measured by TEM. Additionally, the hydration diameter of BSA-Dox-MONPs (370.12 ± 3.34 nm) is higher than Dox-MONPs (313.03 ± 4.67 nm), confirming the success of BSA coating (Figure 4A). Zeta potential measurements showed that MONPs were negatively charged (−18.99 ± 0.86 mV) and switched to positive (40.88 ± 1.99 mV) after Dox loading. After BSA coating, the zeta potential of BSA-Dox-MONPs plummeted to −21.70 ± 0.81 mV because the BSA were negatively charged (Figure 4B). These results suggested the Dox and BSA was successfully loaded on the MONPs. After being stored for 5 days, BSA-Dox-MONPs’ Zeta potential is −22.11 ± 0.96 mV, and the hydration diameter is 368.02 ± 8.90 nm, indicating good stability.

To determine the biocompatibility of MONPs, human keratinocytes HaCaT were incubated with various concentrations of MONPs for 24 h and followed by MTT assays. As shown in Supplementary Figure 3, minimal cell death was observed despite the increase of the concentration of MONPs up to 1 mg/mL, indicating the superior biocompatibility of MONPs.

**FIGURE 5:**
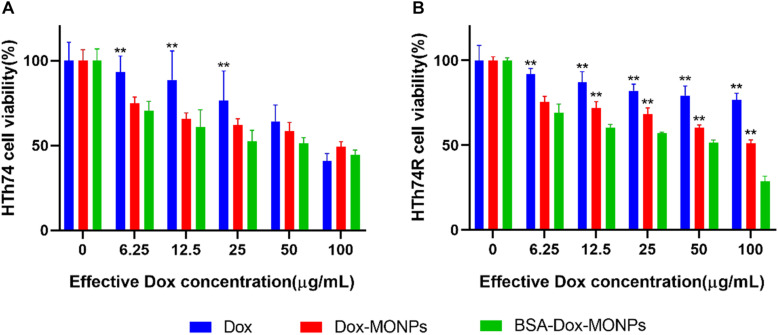
Cell viability of **(A)** HTh74 and **(B)** HTh74R after incubation with various concentrations of MONPs, Dox-MONPs and BSA-Dox-MONPs for 24 h, respectively. *P < 0.05, **P < 0.01, comparison to BSA-Dox-MONPs.

To investigate the impact of MONPs on Dox’s therapeutic efficacy, the viability of Dox-MONPs treated and BSA-Dox-MONPs treated ATC cells was compared at equivalent Dox concentration. As shown in Figure 5 and Supplementary Figure 4, at all free Dox concentrations, the viability of HTh74R was significantly lower than HTh74, indicating that HTh74R was drug-resistant. Moreover, the viability of HTh74R at all concentrations and HTh74 at low concentrations treated with Dox-MONPs was substantially lower than with free Dox at equivalent Dox concentration. After coated with BSA, BSA-Dox-MONPs elicited more significant efficacy than Dox-MONPs to HTh74 and HTh74R, suggesting that the BSA coating further improves the therapeutic effect of Dox-MONPs. Furthermore, the modification of MONPs enhanced the efficacy of drug-resistant HTh74R more than that of sensitive HTh74. It is noted that BSA-Dox-MONPs induced significant cell toxicity to HTh74R at a concentration of 100 μg/mL, indicating that BSA stabilization reversed chemotherapy resistance of ATC.

**FIGURE 6:**
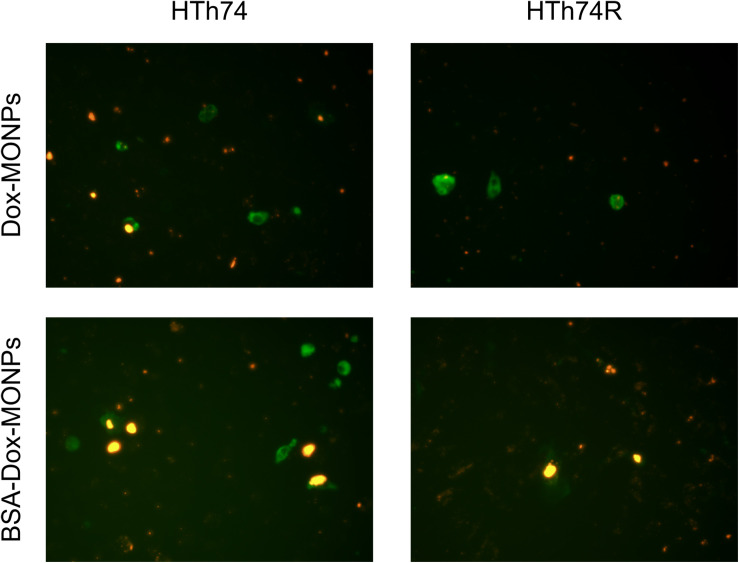
The fluorescence microscope images of YF488-AnnexinV fluorescence staining with HTh74 and HTh74R after incubation with Dox-MONPs and BSA-Dox-MONPs at equivalent Dox concentration of 25 μg/mL for 6 h. The green fluorescence represents early apoptosis and the red fluorescence represents the drug loaded nanoparticles.

Based on the encouraging results of the above-described MTT assay, fluorescence imaging was used to visualize the anti-tumor effect of Dox-MONPs and BSA-Dox-MONPs. HTh74R cells have weaker red fluorescence than HTh74 cells, which further proves to be drug-resistant cells. Yellow signal, which is the overlap of green and red fluorescent, suggested that the cells that ingested nanoparticles were undergoing apoptosis (Figure 6). The overlay image further revealed that drug-loaded nanoparticles promoted early apoptosis of ATC cells. In detail, BSA-Dox-MONPs exhibited stronger yellow fluorescence than Dox-MONPs, suggesting more vital killing ability.

**FIGURE 7:**
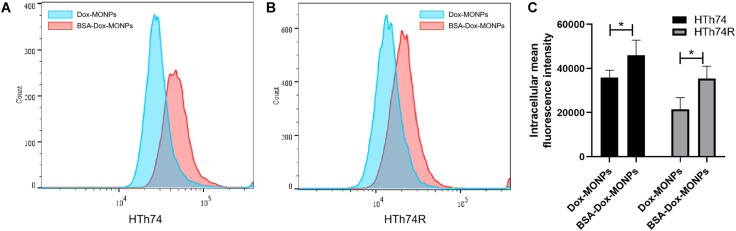
Intracellular Dox fluorescent signal of cells incubated with Dox-MONPs and BSA-Dox-MONPs for 6 h in **(A)** HTh74 and **(B)** HTh74R cells. **(C)** Intracellular fluorescence intensity in HTh74 and HTh74R cells incubated with Dox-MONPs and BSA-Dox-MONPs for 6 h. *P < 0.05.

Insufficient intracellular accumulation of drugs due to inefficient drug uptake and enhanced drug efflux is one of the fundamental mechanisms behind the chemo-resistance of ATC. Thus, to explore the mechanism of the enhanced efficacy of BSA-Dox-MONPs, flow cytometry was employed to determine their uptake and efflux. The fluorescence-activated cell sorting (FACS) results showed that HTh74 internalized drug-loaded nanoparticles more effectively than HTh74R, which is in line with the resistance of HTh74R. As shown in Figure 7, with Dox loaded and BSA coated, it can be visually observed that the FACS curve shifts to the right, suggesting that both HTh74 and HTh74R internalized the drug-loaded nanoparticles. After covering Dox-MONPs with BSA, the fluorescence intensity in HTh74 and HTh74R cells increased by 28.14 and 65.53%, respectively, indicating that BSA can effectively promote the drug uptake by ATC cells. The fluorescence images of HTh74 and HTh74R cells after incubation were also obtained. The images (Supplementary Figures 5, 6) showed greater intracellular accumulation of Dox for BSA-Dox-MONPs in both HTh74 and HTh74R cells, which further confirmed the observation from FACS. Based on the above results, the BSA-stabilized MONPs killed cancer cells more effectively than Dox-MONPs, which may be partly attributed to the enhanced Dox internalization in drug-resistant ATC cells (Yu et al., 2011).

**FIGURE 8:**
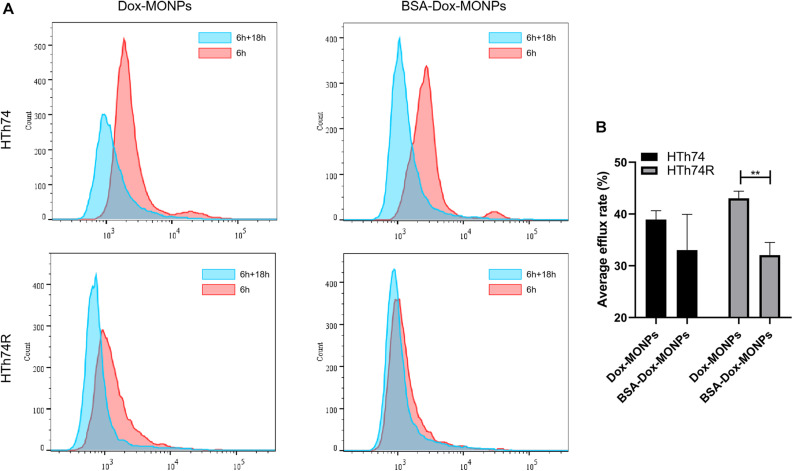
**(A)** Intracellular Dox fluorescent signal of HTh74 and HTh74R cells incubated with Dox-MONPs and BSA-Dox-MONPs for 6 h and incubated with culture medium for another 18 h, respectively. **(B)** The efflux ratio of drug-loaded nanoparticles. **P < 0.01.

To quantify the efflux of the drug, HTh74 and HTh74R were incubated with Dox-MONPs and BSA-Dox-MONPs for 6 h and cultured in fresh medium for another 18 h to allow drug efflux. The efflux rate was defined as the ratio of intracellular fluoresce intensity before and after culture in fresh medium. As shown in Figure 8, the efflux rates of Dox-MONPs and BSA-Dox-MONPs were 38.95 and 33.05% in HTh74 cells and 43.03 and 32.07% in HTh74R cells. It is worth noting that the BSA coating reduced the efflux rate of MONPs, especially in drug-resistant HTh74R. Taken together, these results indicated BSA-Dox-MONPs reversed the resistance of HTh74R cells by enhancing drug uptake and inhibiting drug efflux.

## Conclusion

In summary, we have constructed a BSA-coated MONPs as Dox carrier with high loading efficiency and capacity. The BSA-Dox-MONPs showed stronger cancer-killing power than free Dox and Dox-MONPs, especially for drug-resistant HTh74R cells. This improved therapeutic efficacy can be attributed to enhanced drug uptake and reduced drug efflux of drug-resistant ATC cells. In brief, BSA-Dox-MONPs increased the intracellular accumulation of Dox in drug-resistant ATC cells and thus reversed their chemotherapy resistance via increased drug uptake and inhibited drug efflux, offering a promising platform for the treatment of chemo-resistant ATC.

## Data Availability Statement

The original contributions presented in the study are included in the article/Supplementary Material, further inquiries can be directed to the corresponding author/s.

## Author Contributions

SW presented the initial idea for the research and provided financial support. XH conducted experiments and wrote most of the article. XX analyzed most of the experimental data and wrote part of the article. YTa guided material synthesis and characterization experiments. FZ and YTi gave support on cell experiments and advice on fluorescence imaging. WL and DH analyzed some of the experimental data and helped with the flow cytometry test. SW, GL, and YG reviewed the data and the results of the experiment and gave valuable suggestions for revising the article. All authors contributed to the article and agreed with the submission version.

## Conflict of Interest

The authors declare that the research was conducted in the absence of any commercial or financial relationships that could be construed as a potential conflict of interest.
