# Supplementary material for: BSA-Stabilized Mesoporous Organosilica Nanoparticles Reversed Chemotherapy Resistance of Anaplastic Thyroid Cancer by Increasing Drug Uptake and Reducing Cellular Efflux
Source: Front Mol Biosci. 2020 Dec 3;7:610084. doi: 10.3389/fmolb.2020.610084 (PMC7744685; doi:10.3389/fmolb.2020.610084)
Supplement: Supplementary file 1 [file Table_1.DOCX]

Supplementary Material

1 Supplementary Figures


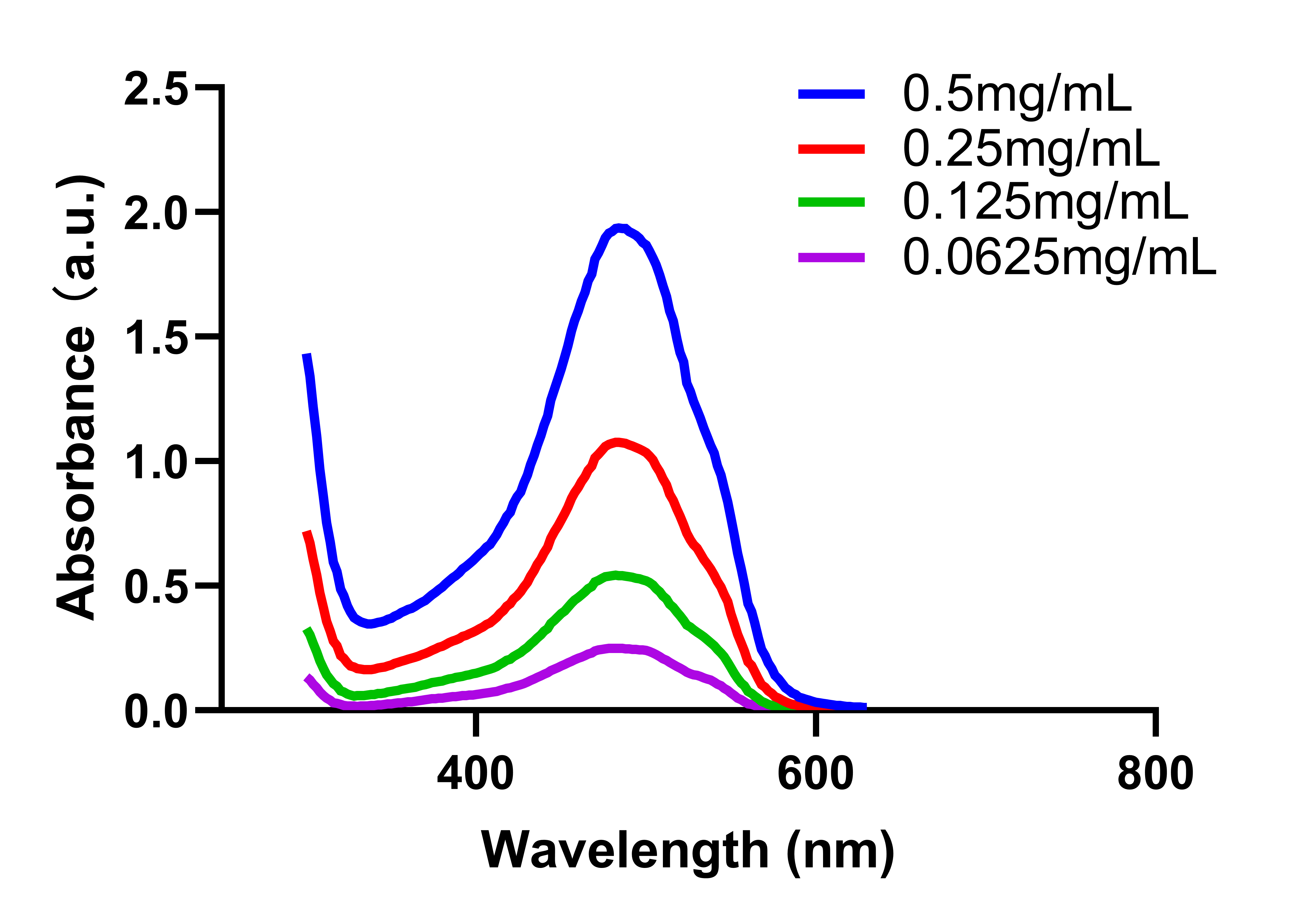


**Supplementary Figure 1.** The UV-Vis spectra from 300 nm to 600 nm of Dox aqueous solution at different concentrations.


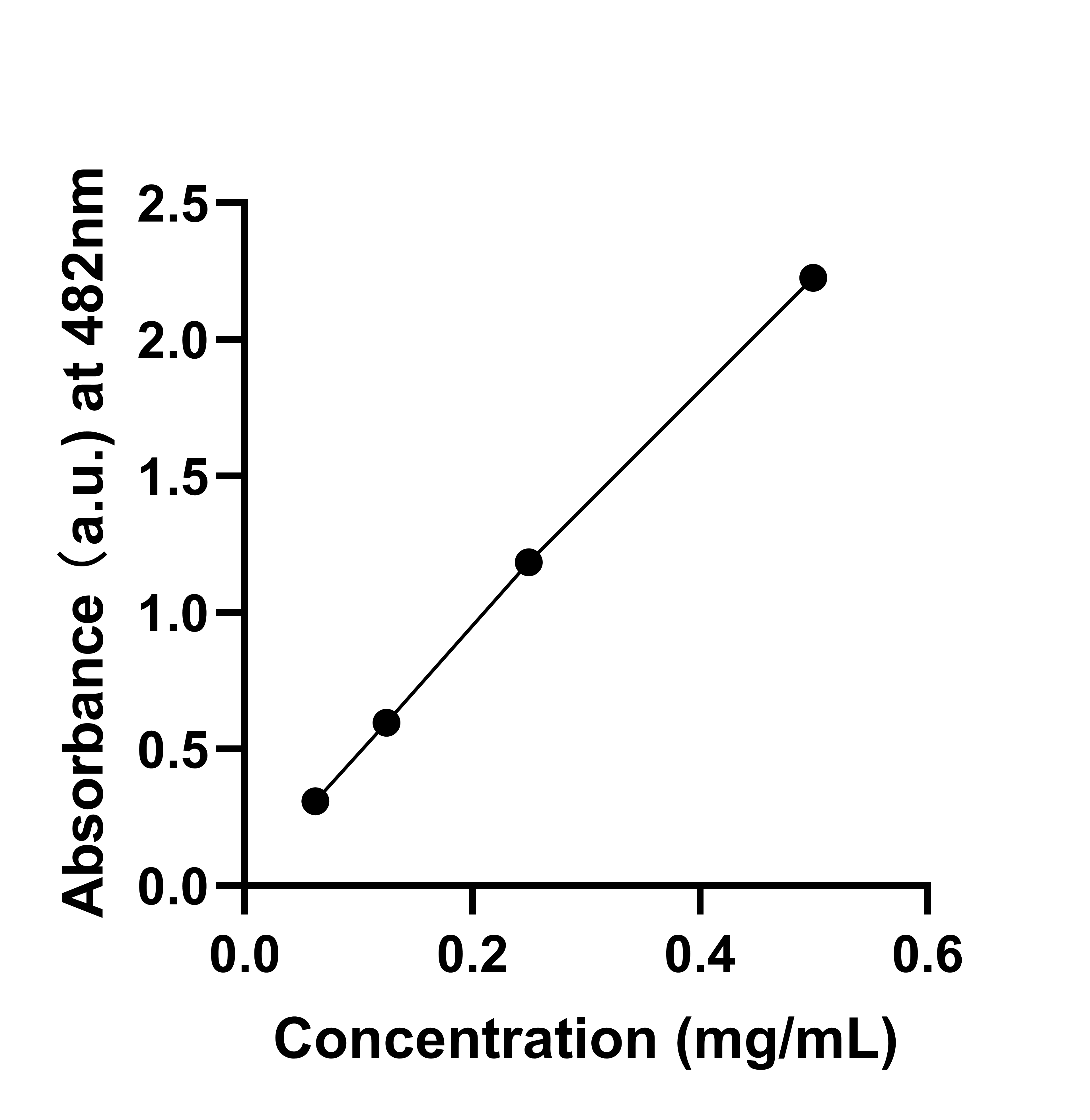


**Supplementary Figure 2.** Concentration standard curve of Dox at 482nm.


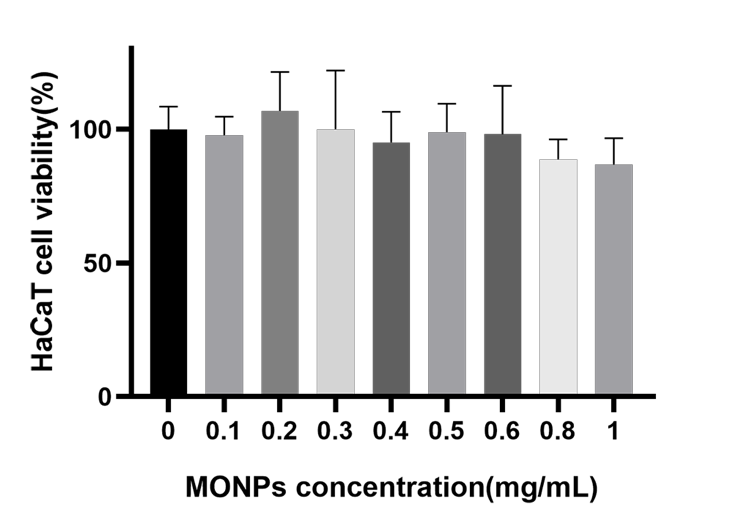


**Supplementary Figure 3.** Cell viability of HaCaT after incubation with various concentrations of MONPs for 24 h.


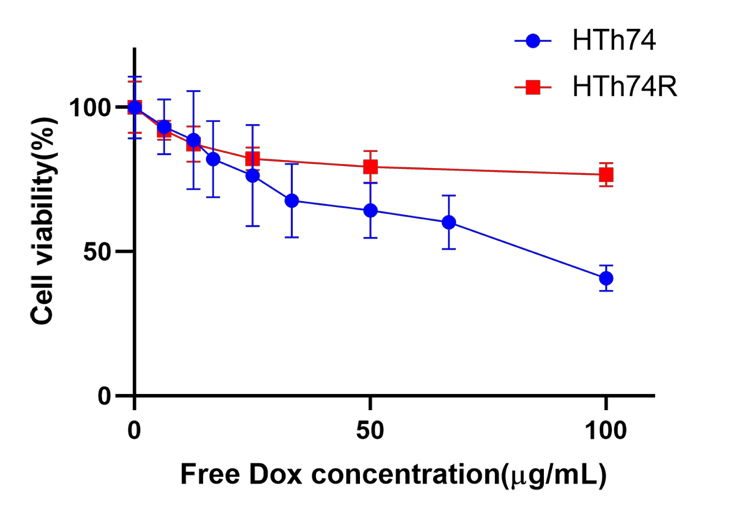


**Supplementary Figure 4.** Cell viability of HTh74 and HTh74R after incubation with various concentrations of free Dox for 24 h.


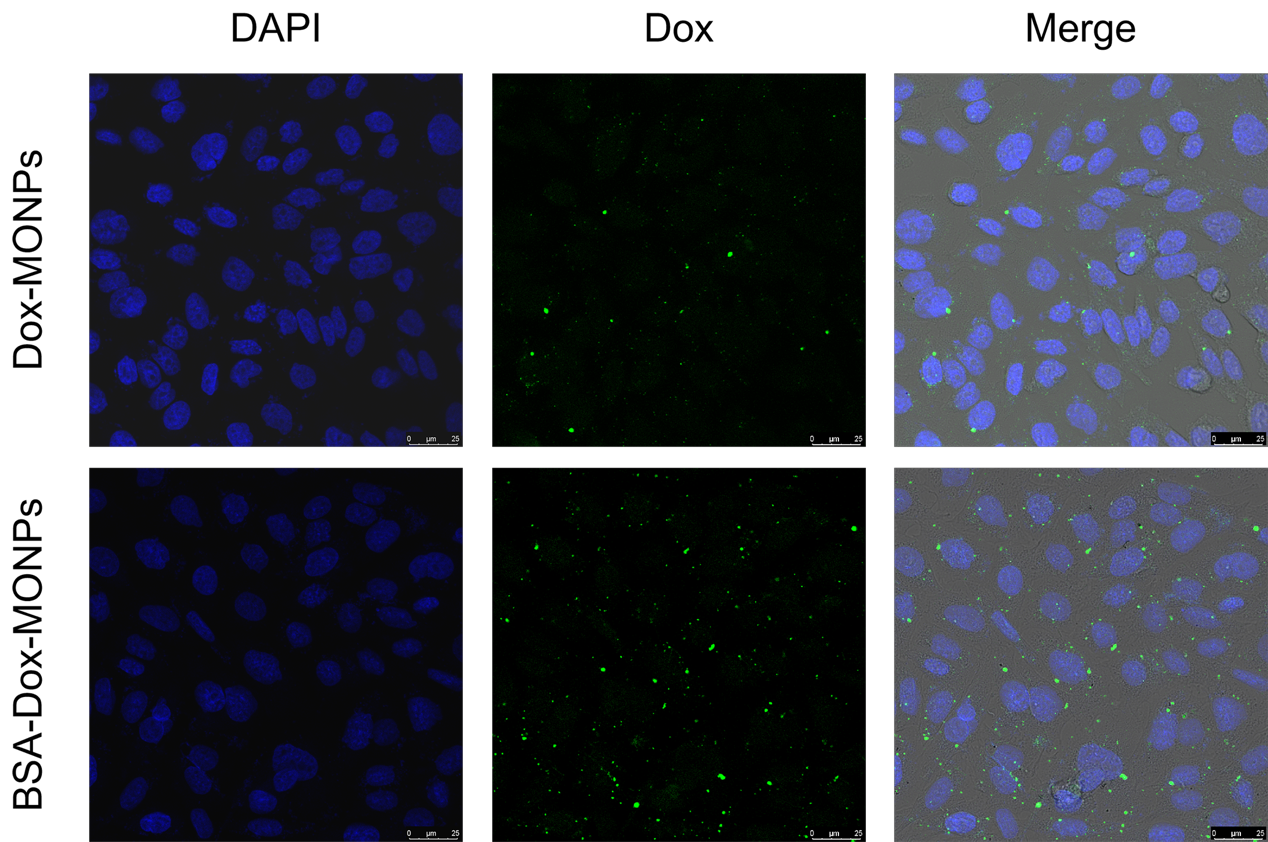


**Supplementary Figure 5.** The confocal images of DAPI staining with HTh74 after incubation with Dox-MONPs and BSA-Dox-MONPs at equivalent Dox concentration of 25 μg/mL for 6 h.


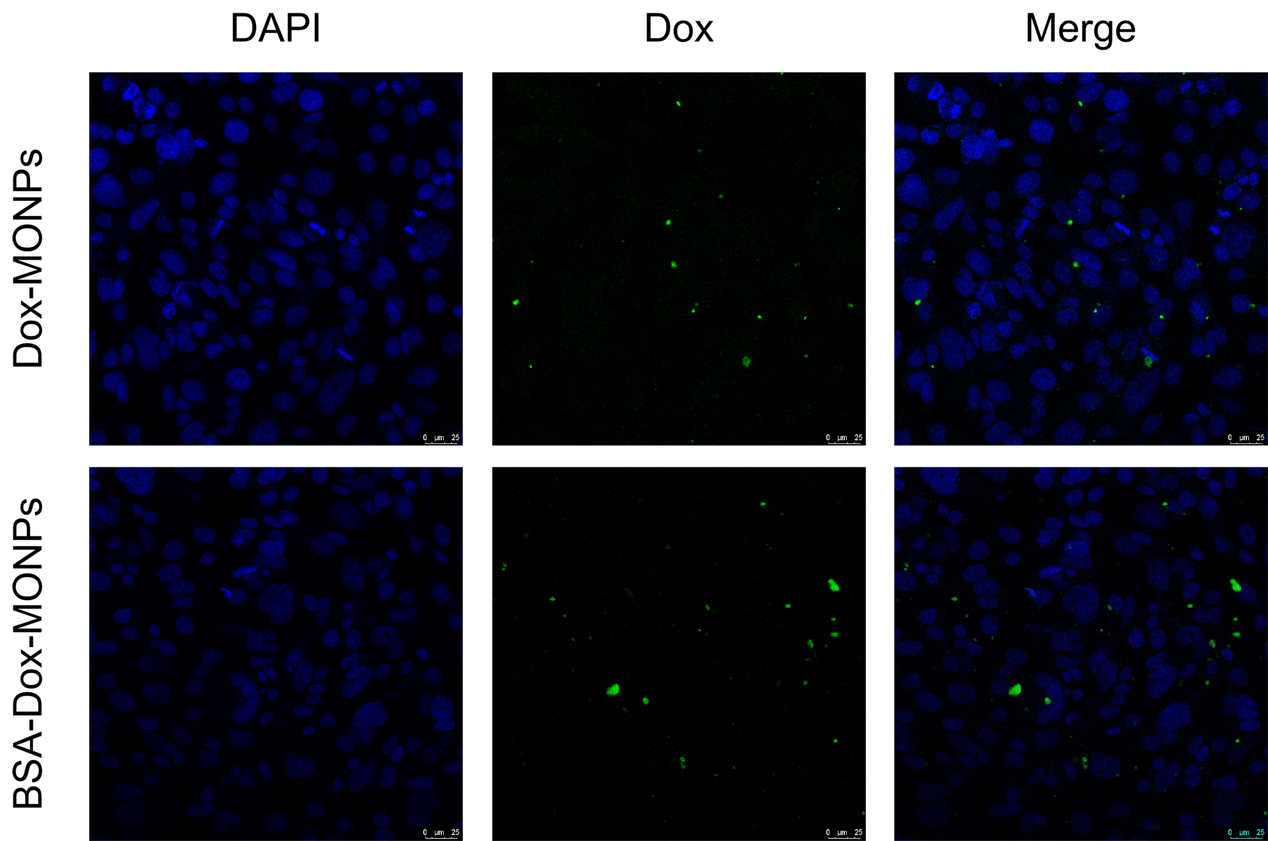


**Supplementary Figure 6.** The confocal images of DAPI staining with HTh74R after incubation with Dox-MONPs and BSA-Dox-MONPs at equivalent Dox concentration of 25 μg/mL for 6 h.
